# Unveiling the nutraceutical potential of indigenous and exotic eggplant for bioactive compounds and antioxidant activity as well as its suitability to the nutraceutical industry

**DOI:** 10.3389/fpls.2025.1451462

**Published:** 2025-02-04

**Authors:** Yvonne Angel Lyngdoh, Partha Saha, Bhoopal Singh Tomar, Rakesh Bhardwaj, Laxman L. Nandi, Mohita Srivastava, Bishal Gurung, Jeetendra Kumar Ranjan, Paresh Chaukhande

**Affiliations:** ^1^ Division of Vegetable Science, ICAR-Indian Agricultural Research Institute, New Delhi, India; ^2^ Regional Station, ICAR – Central Potato Research Institute, Shillong, Meghalaya, India; ^3^ Division of Crop Improvement, ICAR-National Institute for Research on Commercial Agriculture, Research Station, Dinhata, Cooch Behar, West Bengal, India; ^4^ Germplasm Evaluation Division, ICAR- National Bureau of Plant Genetic Resources, New Delhi, India; ^5^ Department of Horticulture, Lovely Professional University, Phagwara, India; ^6^ Department of Statistics, North-Eastern Hill University, Shillong, Meghalaya, India

**Keywords:** eggplant, bioactive compounds, antioxidant activity, principal component analysis, nutraceuticals

## Abstract

Eggplant is a nutritionally rich crop that has beneficial effects on human health. Wide diversity exists in eggplant in terms of biochemical content; however, extensive research work has not been undertaken to gain more in-depth knowledge on its antioxidant capacity to improve the quality of the existing popular cultivated varieties and develop/identify nutrient-rich germplasms. Therefore, a total of 57 genotypes were chosen for the study, and they were evaluated for various biochemical compounds. The biochemical traits taken were an average of three replications and these values were used for statistical analysis. The analysis of variance for five fruit quality parameters indicated a high variability among the eggplant genotypes, which signifies that at least one of the genotypes is statistically different from the rest. The total phenolics ranged from 39.63 to 312.65 mg gallic acid equivalent (GAE)/100 g with the highest being observed in Pusa Krishna. The flavonoid content ranged from 7.83 to 65.09 mg/100 g FW. The antioxidant assays, viz., cupric reducing antioxidant activity (CUPRAC) and ferric reducing antioxidant power (FRAP), were evaluated which ranged from 51.48 to 200.36 mg GAE/100 g for CUPRAC and 35.36 to 214.01 mg GAE/100 g for FRAP. Principal component analysis (PCA) generated a total of five principal components, and the maximum variance of 90.72% was exhibited by the first three PCs. The agglomerative hierarchical clustering (AHC) revealed similar results to the PCA by identifying three major clusters. Cluster 1 had a maximum number of genotypes grouped together, i.e., 48, followed by cluster 2 with six genotypes, viz., Pusa Krishna (G-32), G-5, Mayurbhanj Local, HABI-2, *Solanum gilo*, and *Solanum sisymbrifolium*, and cluster 3 had only three genotypes, namely, *Solanum insanum*, *Solanum khasianum*, and *Solanum xanthocarpum*. Furthermore, the wild species *S. insanum*, *S. khasianum*, and *S. xanthocarpum* can also be utilized as a donor line for carrying out the nutritional breeding program as they are the reservoir of many important biochemical genes.

## Introduction

Diversifying our food basket with fruits and vegetables of different colors not only finds its place as a consumer preference but also helps us consume natural antioxidants, protecting the human body from a variety of chronic and degenerative diseases (Hassan and Abdel-Aziz, 2010). The United States Department of Agriculture (USDA) has recommended the inclusion of fruits and vegetables in our daily diet as the natural pigments present in them are beneficial for human health (Ames et al., 1993). Fruits and vegetables we consume daily in our diet are rich sources of biochemical compounds such as phenolics, including flavonoids and anthocyanin, ascorbic acid, α-tocopherol, β-carotenoids, and lycopene (Kaur et al., 2014). There are lines of evidence confirming the significance of plant phenolics on the human body, viz., anti-inflammatory, antiviral, antibacterial, anti-allergic, antithrombotic, anti-atherosclerotic, and anticarcinogenic properties (Kaur et al., 2014; Koley et al., 2019). Flavonoids are also potent antioxidants that help treat inflammatory conditions, cardiac debility, and hypolipidemia (Mutalik et al., 2003). The colorful appearance of fruits and vegetables due to anthocyanin plays an active role in their antioxidant properties, protecting the DNA as well as plants against cold stress and ultraviolet-induced damage and reducing the risk of many chronic illnesses, particularly cancer (De Pascual-Teresa et al., 2010).

Eggplant is a nutritionally rich crop compared to other vegetables and ranks among the top 10 vegetables for its superoxide scavenging activity (Naeem and Ugur, 2019). However, variability also exists in terms of quality. Eggplant is one of the vegetables having the highest antioxidant capacity, owing to its high phenolic content (Singh et al., 2009), with a meager caloric value, and is considered one of the healthiest vegetables for its high content of vitamins, minerals, and bioactive compounds for human health (Plazas et al., 2013; Docimo et al., 2016). The importance of eggplant for its nutraceutical properties is gaining momentum worldwide among researchers and is regarded as a model crop for carrying out studies for enhancement in quality. The antioxidant capacity in eggplant is very high due to the presence of phenolics, particularly chlorogenic acid (CGA), which is the main phenolic compound (Mennella et al., 2012; Plazas et al., 2013; Naveed et al., 2018).

Eggplant is the third most consumed edible *Solanum* and the most popular solanaceous vegetable widely grown in India and many other countries owing to its variability and diversity in shape, color, and biochemical properties (Yadav et al., 2020). It is commonly known as brinjal, aubergine, melanzana, and garden egg. It is widely used in different food preparations and is therefore known as the king of vegetables (Daunay and Janick, 2007). India stands second in area and production after China with 12.8 million tons of production from an area of 0.73 million hectares and average productivity of 17.5 tons per hectare (Horticultural Statistics at a Glance, 2018). There are also some biotic and abiotic factors that affect the yield and productivity of eggplant. The major problem in terms of biotic stresses is diseases, viz., *Fusarium* wilt, *Phomopsis* blight, root-knot nematode, and viruses, posing a major threat to eggplant cultivation affecting its yield and quality (Elbasuney et al., 2024; Attia et al., 2024). Many research works have also been carried out by various researchers to address these issues (Elbasuney et al., 2024; Abdelaziz et al., 2022; Attia et al., 2024). It is an indigenous crop of India as evident from the reported diversity owing to its wide range of fruit shapes and colors, from oval or egg-shaped to long club-shaped and from white, yellow, and green to degrees of purple pigmentation to almost black and grown in different parts of the country, viz., northeast region and central and eastern India, with approximately 100 local cultivars being grown in West Bengal and Odisha (Devi et al., 2015; Bhanushree et al., 2019; Santhiya et al., 2019; Nandi et al., 2021). In eggplant, diversity not only exists in terms of shape and color but also for various bioactive compounds. However, not much research work has been undertaken in the past to gain more in-depth knowledge and get detailed information on its antioxidant capacity so as to improve the quality of the existing popular cultivated varieties and to develop/identify nutrient-rich germplasms. Hence, understanding the amount and kind of genetic variation within a crop species is a basic prerequisite for initiating and conducting an efficient breeding program. Lately, in the current scenario, breeders have emphasized developing nutritionally rich colored varieties exploring their antioxidant potential. With this knowledge in mind and seeing the potential of eggplant germplasm in terms of biochemical content, the present study was undertaken to evaluate 57 genotypes of eggplant including wild species, popular varieties, and advanced breeding lines for different biochemical contents, viz., total phenolics, antioxidant assay [cupric reducing antioxidant activity (CUPRAC) and ferric reducing antioxidant power (FRAP)], total flavonoid content, and ascorbic acid, and to understand the correlation between these parameters using the chemometric tools like principal component analysis (PCA) and agglomerative hierarchical clustering (AHC). The identification of germplasm rich in biochemical compounds and antioxidant assay will help promote these cultivars in different agroecological regions of India and the world, thereby expanding their accessibility to the local population. Therefore, information generated on the nature and degree of variability and genetic information of the genotypes for biochemical content from this study will enable eggplant breeders to carry out significant research work in nutritional breeding to identify biochemically rich lines and their utilization in further crop improvement.

## Materials and methods

In this study, 57 diverse genotypes of *Solanum melongena* L. comprising commercial varieties, advanced breeding lines, and wild *Solanum* accessions maintained as a pure line at the Division of Vegetable Science, ICAR-Indian Agricultural Research Institute (IARI), New Delhi, India, were incorporated. The seeds were sown in June and 1-month-old healthy seedlings were transplanted at a spacing of 75 × 60 cm with 20 plants in each accession. The experimental design was a randomized block design with three replications. The recommended packages of practices were followed as per standard package of practices to raise a healthy crop. During the growing period (June to November), the average temperature was 28°C with 72% relative humidity and an average wind speed of 3.8 km per h.

### Estimation of bioactive compounds

Three mature edible fruits were harvested randomly based on the color and glossiness of the fruit skin of the particular accession. The fruits were collected in the morning hours from the middle of each plant, and there was no harvesting from the border plants. Healthy, injury-free fruits were then taken to the laboratory, and after thorough cleaning and washing, they were further subjected to biochemical analysis.

### Extraction of the homogenate

The fresh fruits of brinjal weighing 5 g were cut into small pieces, and 10 ml of 80% ethanol was added to it and left overnight. The next day, the sample was crushed using a pestle and mortar. The homogenate was centrifuged at 5,000 rpm for 20 min. The supernatant was collected in a separate container; the residue was re-extracted at least two more times with 5 ml of 80% ethanol and centrifuged, and the supernatants were pooled together. The final volume was made to 25 ml in a volumetric flask with 80% ethanol after filtering in Whatman filter paper grade 42.

### Total phenolics

The total phenolic was estimated spectrophotometrically following the procedure of Singleton et al. (1999) by using Folin–Ciocalteau as the reagent (FCR). One hundred microliters (0.1 ml) of the sample aliquots were taken in a test tube and allowed to dry in a boiling water bath. The test tubes were removed once the sample content dried and was allowed to cool down. To the test tube, 3 ml of distilled water was added and the contents were dissolved by vortex mixing followed by adding 0.5 ml of FCR reagent. After 3 min, 2 ml of 20% Na_2_CO_3_ solution was added to the test tubes and vortexed once again to mix the sample thoroughly and kept in the dark for 60 min. The absorption was read at 750 nm against a reagent blank in a UV–vis spectrophotometer. A calibration curve was drawn with standard gallic acid and expressed as gallic acid equivalent (mg GAE/100 g fresh weight).

### Total flavonoid content

The total flavonoid content was estimated as per Zhishen et al. (1999). One milliliter of the sample aliquot was extracted in 10 ml of a volumetric flask containing 4 ml of distilled water, 0.3 ml portion of 5% NaNO_2_, and 0.3 ml portion of 10% AlCl_3_·6H_2_O. The mixture was kept at room temperature for 6 min followed by adding 2 ml of 1 mol/L NaOH and diluting it to 10 ml of distilled water. The absorbance was immediately read at 510 nm wavelength, and the results were expressed as mg/100 g FW.

### Antioxidant assay

#### Cupric reducing antioxidant capacity

The antioxidant assay CUPRAC was executed by following the procedure of Apak et al. (2004). The main principle was the absorbance measurement of the CUPRAC chromophore, Cu (I)-neocuproine (Nc) chelate, formed as a result of the redox reaction of antioxidants with the CUPRAC reagent, bis(neocuproine) copper (II) cation [Cu(II)-Nc], and absorbance was read at the maximal light absorption wavelength of 450 nm. A total of 500 µl (0.5 ml) of the aliquot sample was taken in the test tube, and the volume was made up to 1,100 µl with distilled water. This was followed by adding 1 ml each of ammonium acetate buffer (pH 7), followed by CuCl_2_ and then finally neocuproine to make the final volume of 4.1 ml. The test tubes were appropriately shaken by vortexing and incubated for 30 min. Finally, absorbance was measured at 450 nm in a spectrophotometer. Antioxidant activity was calculated from the standard curve and expressed as mg GAE 100 g/FW.

#### Ferric reducing antioxidant power assay

The antioxidant assay FRAP was executed by following the procedure of Benzie and Strain (1996) with the principle based on the conversion of ferric tripyridyl triazine [Fe (TPTZ)]^3+^ complex to its ferrous [Fe (TPTZ)]^2+^, which forms an intense blue color at 593 nm in the presence of antioxidants. First, 100 µl (0.1 ml) of the sample aliquot was taken in the test tube followed by adding 200 µl (0.2 ml) of distilled water. This was followed by the addition of FRAP reagent 2,200 µl which was prepared freshly before using the test tubes. The FRAP reagent is a combination of 0.1 M acetate buffer having a pH of 3.6, TPTZ 10 mM in 40 mM of HCl and 20 mM of FeCl_3_.6H_2_O, in the ratio of 10:1:1. Then, the tubes were vortexed for a proper mixture of the contents followed by incubation for 30 min. Finally, the absorbance was read at 593 nm in a UV–vis spectrophotometer. The standard curve was calibrated using gallic acid as the standard, and the results obtained were expressed as mg GAE 100 g/FW.

#### Ascorbic acid

Ascorbic acid content was estimated spectrophotometrically following the procedure of Jagota and Dani (1982). First, a fresh sample (2 g) was weighed and ground with 6% metaphosphoric acid–EDTA solution, and the volume was made up to 50 ml with 4.5% metaphosphoric acid. The above mixture was then centrifuged at 5,000 rpm for 10 min and filtered through a Whatman No. 1 paper, and the supernatant was collected in a volumetric flask. The sample aliquot (1.2 ml) was then taken in a test tube and the volume was made up to 4 ml with distilled water and mixed well. Next, 0.4 ml of Folin–Ciocalteau reagent was added to each test tube and then incubated for 10 min at room temperature. This was followed by centrifugation at 3,000 rpm for 10 min. The clear supernatant solution was collected after centrifugation and read against a blank solution in a spectrophotometer at 760 nm. For making the standard curve, 100 mg of ascorbic acid was dissolved in 100 ml of 3% metaphosphoric acid from which 10 ml was taken and further diluted to 100 ml of 3% metaphosphoric acid to prepare the working standard solution. The concentration of ascorbic acid in the sample was calculated from the slope of the ascorbic acid standard curve and expressed as mg of ascorbic acid 100 g/FW.

### Statistical analysis

The 57 genotypes and five biochemical parameters (total phenolics, CUPRAC, FRAP, total flavonoid, and ascorbic acid) were subjected to data analysis in a matrix order of 57 × 5. The biochemical traits taken were an average of three replications, and these values were used for statistical analysis of variance as per the procedure given by Panse and Sukhatme (1967). Tukey’s test was further used to investigate the differences among the genotypes at *p ≤*0.01 using SAS^®^ 9.2 software. Estimation of parameters of variability and genotypic and phenotypic coefficient of variation was done following Burton and De Vane (1953) and broad sense heritability by Falconer (1981), and the expected genetic advance (GA) was calculated as per Johnson et al. (1955).

PCA, a multivariate statistical tool, was employed for data reduction. PCA captures patterns in data expressing them in such a way as to identify similarities and differences (Granato et al., 2010). The AHC was employed to study the possibility of classifying brinjal genotypes based on their similarities for the different compounds (Johnson and Wichern, 2007). The criterion of average distance was used to conduct the AHC procedure. SAS ver. 9.2 and R package were employed for carrying out the entire statistical analysis.

## Results

### Analysis of variance

**Figure 1:**
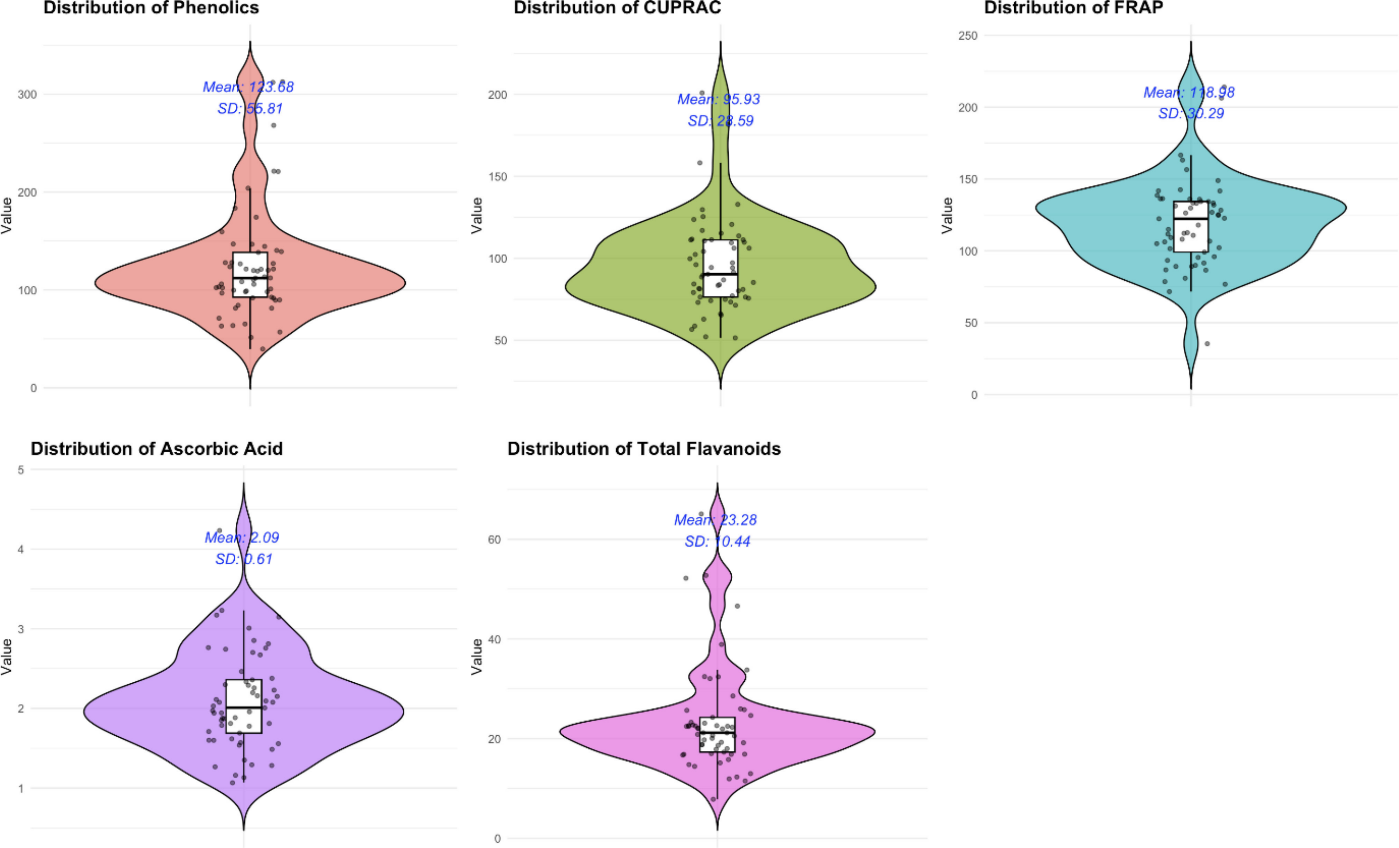
A violin plot showing the distribution of the five different biochemical parameters among the 57 diverse brinjal genotypes.

The analyses of variance for five parameters about the fruit quality of 57 diverse genotypes of *S. melongena* are presented in **Table 1** and **Figure 1**. The mean sum of squares was significant for all parameters, indicating a high variability among the eggplant genotypes, which in turn means that at least one of the genotypes was statistically different from the rest.

**Table 1:** Mean performance of the 57 eggplant accessions for bioactive compounds and antioxidant assay.

Genotypes	Phenolics (mg GAE/100 g)	Total flavonoids (mg GAE/100 g)	CUPRAC (mg GAE/100 g)	FRAP (mg GAE/100 g)	Ascorbic acid (mg/100 g)
Pusa Upkar	140.34	22.76	99.76	93.37	2.23
Pusa Kaushal	81.37	19.27	80.16	138.59	2.33
Pusa Uttam	119.22	25.98	74.20	105.02	3.17
Pusa Shyamla	99.68	18.64	58.69	89.09	2.70
Pusa Kranti	102.15	25.81	62.88	106.73	1.62
Pusa Hybrid 20	89.46	21.16	73.36	80.93	1.81
Pusa Bindu	102.31	14.43	71.38	101.55	1.60
Pusa Purple Cluster	108.43	20.06	75.67	108.08	1.88
Pusa Bhairav	98.13	16.70	56.68	91.51	2.01
Pusa Purple Long	103.15	22.28	83.95	117.94	2.16
Pusa Hybrid 5	91.87	19.76	81.31	106.23	2.03
Pusa Hybrid 9	65.19	22.62	65.38	76.77	2.20
Pusa Hybrid 6	112.08	18.77	66.14	86.77	1.81
Pusa Anupam	121.07	24.26	88.60	135.89	2.30
Pusa Purple Round	121.36	23.28	76.39	128.28	2.76
Pusa Ankur	92.60	16.85	51.48	86.56	2.26
Dinhata Local	98.91	22.47	110.67	133.11	2.08
G-23	110.57	25.68	96.10	129.78	1.28
G-43	81.33	21.96	109.52	141.77	1.56
BB-7	100.99	19.18	86.84	126.84	1.57
G-5	174.25	12.98	91.48	136.08	1.79
G-27	126.97	15.14	73.10	78.58	1.94
Pusa Krishna	221.30	20.85	74.94	89.16	1.07
G-94	119.84	38.91	106.20	136.34	1.88
Pink (reddish)	121.34	16.84	76.38	109.23	1.54
Pinky	63.58	22.48	89.67	112.22	1.29
Pusa Safed Baingan 1	146.92	16.88	83.47	99.11	1.60
HABI-2	220.97	46.60	132.89	166.62	1.49
G-10	112.22	7.83	104.17	163.17	2.46
*S. undatum*	127.96	24.65	113.67	122.67	1.13
Pusa Hara Baingan 1	120.09	22.61	94.41	110.79	2.15
Kushpada Local	51.49	33.79	79.16	89.92	1.35
Guhala Chatua Local	113.30	22.46	81.01	111.66	2.11
Mayurbhanj Local	159.41	32.05	106.21	141.69	1.87
BB-44	56.94	17.00	90.28	102.19	1.96
Hybrid 183	127.83	17.86	97.25	131.25	1.69
G-131	71.07	11.93	81.61	95.42	1.27
EC 368225	126.59	32.40	129.65	156.52	1.78
G-164	91.61	15.80	94.18	124.73	2.85
*S. sisymbrifolium*	183.56	23.10	125.43	125.20	4.23
*S. aethiopicum* Acc 1	106.09	22.55	102.33	112.67	3.15
*S. macrocarpon*	98.12	14.79	109.81	134.31	2.36
*S. aethiopicum* Acc 2	123.70	21.16	115.26	133.29	3.01
*S. anguivi* Acc 1	138.31	17.99	123.67	133.33	2.08
*S. anguivi* Acc 2	144.44	17.30	117.00	126.33	1.97
*S. aethiopicum* Acc 3	96.79	22.00	111.33	122.33	2.76
*S. aethiopicum* Acc 4	105.80	22.12	120.67	148.92	3.23
*S. viarum*	146.69	20.54	111.20	136.29	2.09
*S. xanthocarpum*	312.05	52.76	181.60	142.57	2.38
*S. gilo*	203.92	20.66	111.67	134.33	1.85
*S. khasianum*	268.13	65.09	158.21	214.01	1.94
*S. integrifolium*	39.63	11.51	52.15	35.36	2.67
*S. incanum*	63.07	12.30	85.29	71.63	2.81
*S. insanum*	312.65	52.20	200.86	206.33	2.74
Arka Shirish	139.11	32.45	111.29	131.87	2.29
DB-1	84.28	28.56	84.34	114.97	1.16
G-16	89.78	18.81	77.11	95.95	1.71
Mean	123.68	23.28	95.93	118.98	2.09
SEM (SE)	1.66	0.23	1.36	1.55	0.02
SD	2.86	0.40	2.35	2.68	0.03
CD 0.05	4.69	0.66	3.84	4.39	0.06
CD 0.01	6.24	0.88	5.11	5.84	0.08
CV %	2.32	1.74	2.45	2.26	1.81
Max	312.65	65.09	200.86	214.01	4.23
Min	39.63	7.83	51.48	35.36	1.07

### Total phenolics

In the present study comprising 57 genotypes of different colors and shapes of eggplants, a wide variation was observed for total phenolics, ranging from 39.63 to 312.65 mg GAE/100 g depicting nearly eight-fold variations. Considering only the cultivated genotypes, the total phenolics content ranged from 51.49 to 221.30 mg GAE/100 g with the highest being observed in the variety Pusa Krishna, followed by HABI-2 (220.97 mg GAE/100 g) and G-5 (174.25 mg GAE/100 g), and the lowest being recorded in Kushpada Local (51.49 mg GAE/100 g). Among the wild accessions, *Solanum insanum* (312.65 mg GAE/100 g) had significantly higher phenolic content which was followed by *Solanum xanthocarpum* (312.05 mg GAE/100 g) and *Solanum khasianum* (268.13 mg GAE/100 g).

### Total flavonoid content

Among the germplasms, it was noted that the wild species had more flavonoid content as compared to the cultivated ones, the highest being observed in the wild species *S. khasianum* (65.09 mg/100 g) followed by *S. insanum* (52.20 mg/100 g) and the lowest being observed in *Solanum integrifolium* (11.51 mg/100 g). HABI-2 (46.60 mg/100 g) was the highest among the cultivated genotypes followed by G-94 (38.91 mg/100 g) and the lowest was observed in G-10 (7.83 mg/100 g).

### Antioxidant assay

The results of the antioxidant assay are represented in **Table 1**. Similar to the above two traits, the wild species had significantly higher antioxidant assay than the cultivated ones with a range of 51.48–200.36 mg GAE/100 g for CUPRAC. The maximum CUPRAC activity was observed in *S. insanum* (200.86 mg GAE/100 g) followed by *S. xanthocarpum* (181.60 mg GAE/100 g) and *S. khasianum* (158.21 mg GAE/100 g), and the minimum was recorded in *S. integrifolium* (52.15 mg GAE/100 g). In the cultivated genotypes, the maximum CUPRAC content was observed in HABI-2 (132.89 mg GAE/100 g) followed by EC 368225 (129.65 mg GAE/100 g) and Arka Shirish (111.29 mg GAE/100 g), and the minimum was observed in Pusa Ankur (51.48 mg GAE/100 g). A wide variation was observed for FRAP activity which ranged from 35.36 to 214.01 mg GAE/100 g. FRAP assay was observed to be the highest in *S. khasianum* (214.01 mg GAE/100 g) followed by *S. insanum* (206.33 mg GAE/100 g) and *Solanum aethiopicum* Acc 4 (148.92 mg GAE/100 g) and the lowest in *S. integrifolium* (35.36 mg GAE/100 g) among the wild species, and in the cultivated genotypes, the highest FRAP activity was observed in HABI-2 (166.62 mg GAE/100 g), followed by G-10 (163.17 mg GAE/100 g) and EC 368225 (156.52 mg GAE/100 g), and the minimum FRAP activity was observed in Pusa Hybrid 9 (76.77 mg GAE/100 g).

### Ascorbic acid content

The ascorbic acid content in brinjal genotypes ranged from 1.07 to 4.23 mg/100 g FW, depicting a three-fold variation with a maximum in *Solanum sisymbrifolium* (4.23 mg/100 g) followed by *S. aethiopicum* Acc 4 (3.23 mg/100 g) and *S. aethiopicum* Acc 1 (3.15 mg/100 g). The minimum was observed in *Solanum gilo* (1.85 mg/100 g) among the wild species. Pusa Uttam (3.17 mg/100 g) was observed to be the highest for ascorbic acid content followed by G-164 (2.85 mg/100 g) and Pusa Shyamla (2.70 mg/100 g), and the minimum was observed in G-32 (1.07 mg/100 g).

### Genetic component analysis

In the present study, the variation observed for GCV and PCV was higher than 20% for the traits investigated for different biochemical contents (**Table 2**). However, minute differences between GCV and PCV were observed for total phenolics, flavonoids, CUPRAC, FRAP, and ascorbic acid. High heritability with high genetic advance was observed for all the biochemical traits under study, viz., total phenolics, CUPRAC, FRAP, ascorbic acid, and total flavonoids.

**Table 2:** Estimates of variability, heritability, genetic advance, and GA as percent of mean for bioactive compounds and antioxidant activity.

		Range						
	Mean	Max	Min	GCV	PCV	Broad sense H	GA	GA as % of mean
Total phenolics	123.68	312.65	39.63	45.10	45.16	99.74	114.76	36.71
CUPRAC	95.93	200.86	51.48	29.76	29.86	99.33	58.62	29.19
FRAP	118.98	214.01	35.36	25.43	25.53	99.22	62.08	29.01
Ascorbic acid	2.09	4.23	1.07	29.23	29.28	99.62	1.25	29.67
Total flavonoids	23.28	65.09	7.83	44.85	44.89	99.85	21.49	33.02

### Correlation coefficient

**Figure 2:**
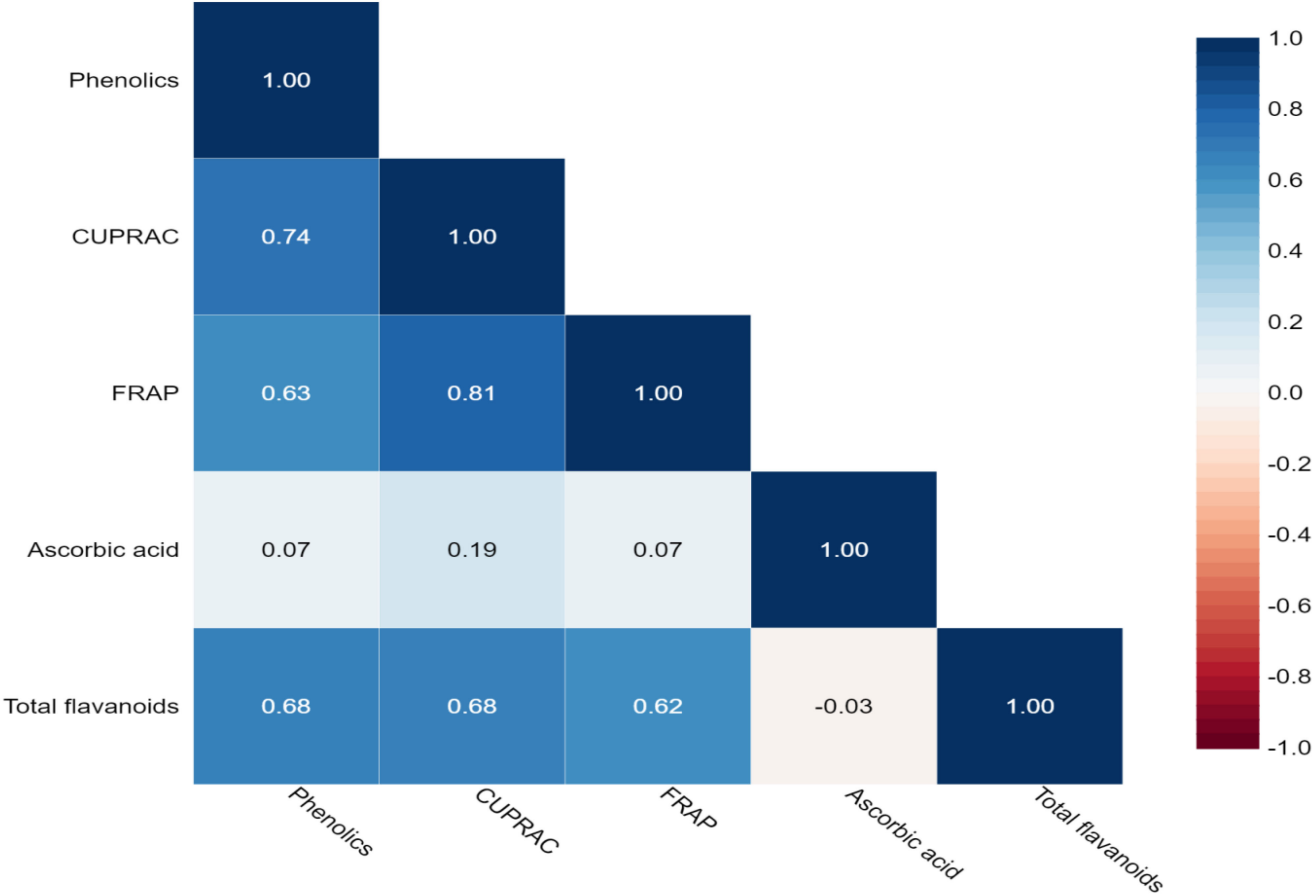
Heatmap showing Pearson’s correlation coefficients for bioactive compounds and antioxidant activity among eggplant accessions. Significant correlations are colored in blue (positive), while correlations that are not significant are in white. Numerical values as shown in **Table 3**.

In the present study, the Pearson correlation coefficient was performed for the bioactive compounds shown in **Table 3** and **Figure 2**. A significant and positive association was observed between the total phenolics with CUPRAC (0.744), FRAP (0.626), ascorbic acid (0.067), and total flavonoids (0.680). This is because total phenolics contributed majorly to antioxidant capacity. Furthermore, CUPRAC had a significant and positive correlation with FRAP (0.811) and total flavonoids (0.684). Similarly, FRAP reported a positive and significant association with total flavonoids.

**Table 3:** Correlation coefficients between bioactive compounds and antioxidant activity among the eggplant accessions.

		Total phenolic	CUPRAC	FRAP	Ascorbic acid	Total flavonoids
Total phenolic	G	1.000				
	P	1.000				
CUPRAC	G	0.744^**^	1.000			
	P	0.743^**^	1.000			
FRAP	G	0.626^**^	0.811^**^	1.000		
	P	0.626^**^	0.810^**^	1.000		
Ascorbic acid	G	0.067	0.192	0.074	1.000	
	P	0.067	0.190	0.074	1.000	
Total flavonoids	G	0.680^**^	0.684^**^	0.620^**^	−0.033	1.000
	P	0.678^**^	0.681^**^	0.618^**^	−0.032	1.000

^**^Significant at the 1% level of significance and significant at the 5% level of significance.

### Principal component analysis

**Figure 3:**
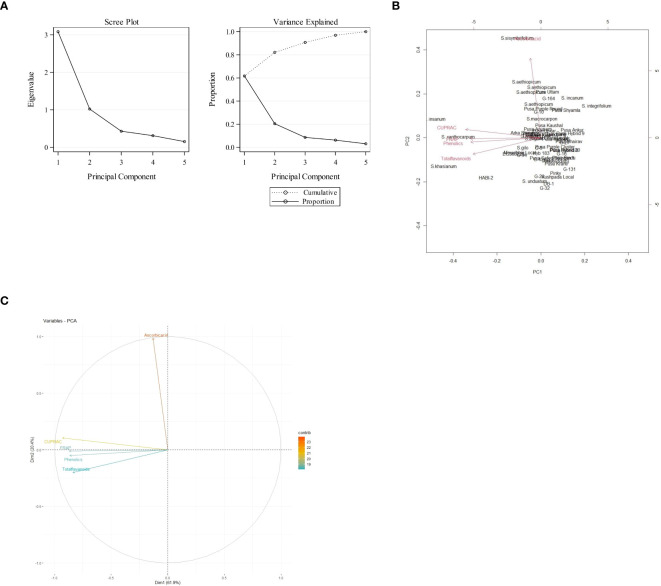
**(A)** Scree plot depicting the eigenvalue of each principal component. **(B)** Percent variance explained by each of the five principal components. **(C)** Variable correlation plot in the first two principal components.

**Figure 4:**
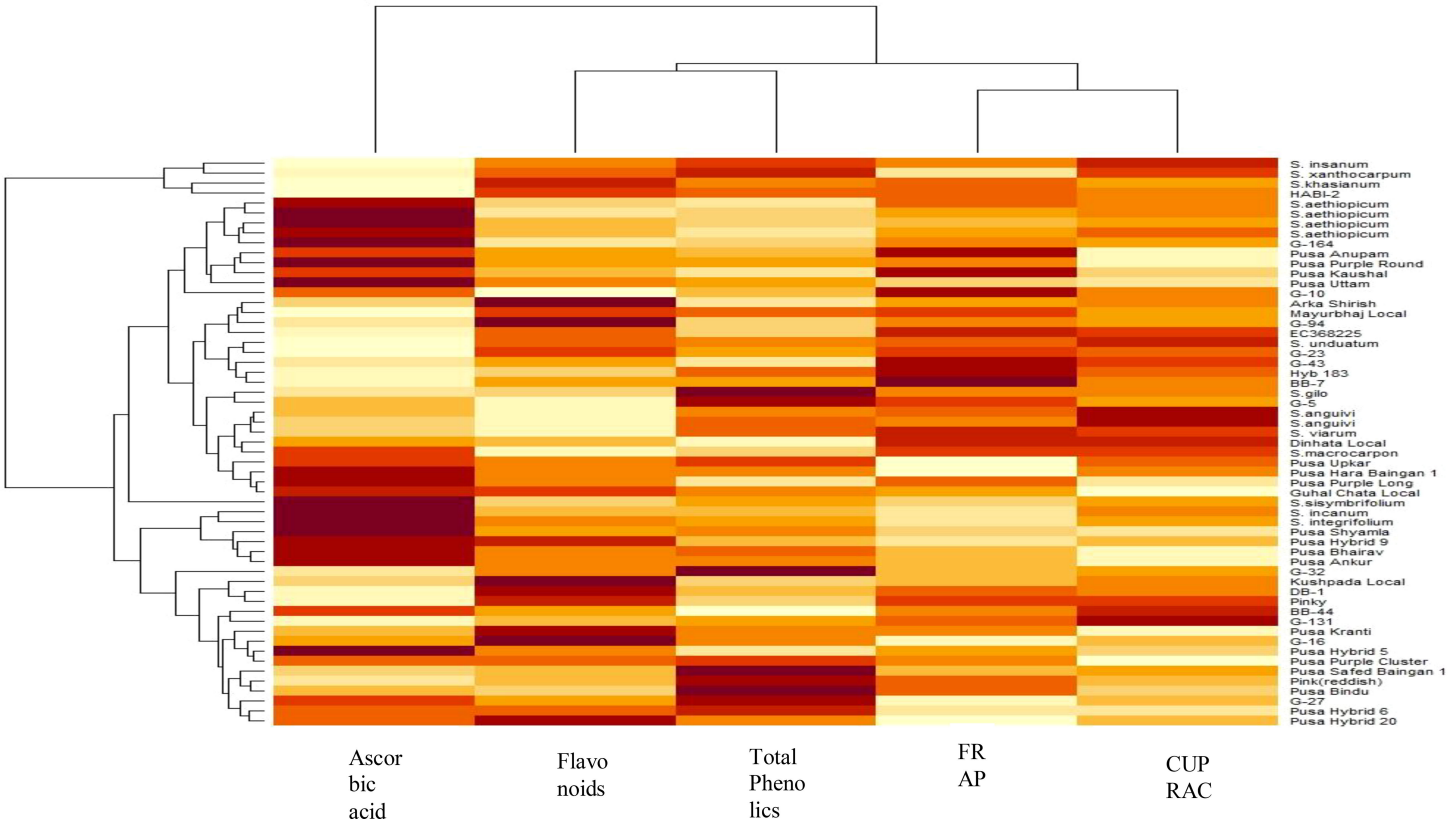
Hierarchical clustering analysis and heatmap of bioactive compounds and antioxidant assay. The color temperature scheme indicates relative variable levels ranging from minimum (white) to maximum (purple) contents of the respective variable.

Data recorded from the 57 eggplant genotypes were subjected to PCA to understand the fundamental constitution of the experiment and the relationship between the eggplant genotypes and the various parameters. In **Figure 3**, the scree plot and percent variance are explained by the principal components. PCA generated a total of five principal components, and the maximum variance (90.72%) was exhibited by the first three PCs. The highest eigenvalue of 3.084 was observed in the first principal component (PC1), which described 61.68% of the variance, and the second principal component (PC2) had an eigenvalue of 1.02 and explained 20.44% of the variance (**Table 4**). The remaining three generated PCs counted less toward total variability. Clusters were formed and explained with a heatmap illustration based on the correlation coefficient between the accessions and the bioactive compounds. In this investigation, the nutritional parameters were also represented in a heatmap (**Figure 2**) which is evident from the range of colors marking the graphical illustration of the individual values in the map. The map also showed that the identical genotypes pertaining to a particular trait were grouped. Three major clusters were identified from this heatmap and the genotypes were grouped according to the respective clusters (**Figure 4**). However, it was observed that the highest number, i.e., 31 genotypes, was grouped in cluster 1. Cluster 2 had 22 genotypes and cluster 3 had only four genotypes, namely, *S. xanthocarpum*, *S. insanum*, HABI-2, and *S. khasianum.* The genotypes present in clusters 2 and 3 are the major contributors to total phenolics and antioxidant assay and can be used in future breeding work for developing hybrids rich in nutraceutical compounds.

**Table 4:** Eigenvalues, % of variance, and cumulative % of bioactive compounds and antioxidant activity among the eggplant accessions.

Principal component	Eigenvalues	% of variance	Cumulative %
1	3.084	61.68	61.68
2	1.021	20.44	82.12
3	0.429	8.60	90.72
4	0.311	6.22	96.94
5	0.153	3.06	100.00

### Agglomerative hierarchical clustering

**Figure 5:**
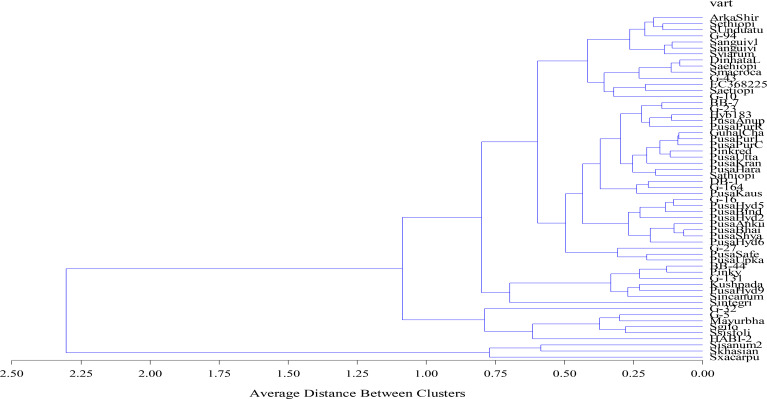
Agglomerative hierarchical clustering (AHC) of 57 eggplant genotypes.

Agglomerative hierarchical clustering is another chemometric tool undertaken to verify the PCA results. This technique involves assembling the genotypes based on uniformity and the result is presented in the form of a dendrogram (**Figure 5**). Similar to the PCA results, the dendrogram identified three major clusters of the 57 genotypes. Cluster 1 had the maximum number of genotypes grouped together, i.e., 48, followed by cluster 2 with six genotypes, viz., Pusa Krishna (G-32), G-5, Mayurbhanj Local, HABI-2, *Solanum gilo*, and *Solanum sisymbrifolium*, and cluster 3 had only three genotypes, namely, *S. insanum*, *S. khasianum*, and *S. xanthocarpum.*

## Discussion

Kwon et al. (2008) conducted a study that indicated that total phenolic content is the main bioactive compound present in eggplant and is responsible for their antioxidant activities (Kwon et al., 2008). The wild accessions under study are rich sources of total phenolics, as similar studies conducted by Kaur et al (Kaur et al., 2014), Nandi et al (Nandi et al., 2021), and Kaushik (2020) indicated that the wild accessions varied significantly in terms of total phenolics. Compared to the wild species, the cultivated genotypes had low phenolic content because of low browning genotypes, and people are not aware of the health properties eggplant possesses (Prohens et al., 2007; Nandi et al., 2021). The total flavonoid content, particularly quercetin, is an important antioxidant with many biological activities beneficial for human health in curing innumerable diseases, viz., cancer, cardiovascular, and neurodegeneration. There was an eight-fold increase in variation in the total flavonoid content ranging from 7.83 to 65.09 mg/100 g FW in the genotypes taken for the study. The wild species are rich reservoirs and storage of flavonoid content, and this is the reason that they are showing high data in comparison to cultivated ones. However, less research has been conducted so far on this aspect, and emphasis should be made on implementing wild types in cultivated genotypes for the transfer of genes and re-establishing the functional pathway for high flavonoid content (Willits et al., 2005), Nandi et al. (2021). also reported similar significant results for flavonoid content in the genotypes which were taken for study. CUPRAC and FRAP are the two crucial antioxidant assays which are needed to screen a large number of genotypes (Kaur et al., 2014). These assays are relatively cheaper, reasonable, and compatible and deliver results without using sophisticated instruments. The wild species, mainly the white- and green-colored fruits, had more phenolics and flavonoids and higher antioxidant activity than the cultivated genotypes observed by Koley et al (2019). The FRAP assay was significantly higher (pb 0.05) than the CUPRAC assay when comparing the antioxidant activity. This could be due to the fact that the mechanism reaction used in both protocols was different. It was also observed that the genotypes that had higher phenolics were also reported to have high flavonoids and antioxidant activity, which was also reported by Koley et al. (2019) and Nandi et al. (2021) . This phenomenon could be due to the phenolic profile differences, i.e., qualitative (type of phenolics) and quantitative (amount of phenolics) present in the different genotypes.

The knowledge of genetic variability that is heritable from generation to generation has great significance in breeding programs, especially in developing new varieties rich in biochemical compounds. The additive, dominance, and epistasis effect govern a crop’s phenotype. For an effective selection to be carried out, variation present in the material is the basic requirement needed which is usually divided into two types, i.e., phenotypic variation (PV) and genotypic variation (GV). However, minute differences between GCV and PCV were observed for total phenolics, flavonoids, CUPRAC, FRAP, and ascorbic acid in the study. This is basically due to the small role played by the environment in the expression of a particular character.

Heritability is of interest to the plant breeder primarily as a measure of the value of section for a particular character in various types of progenies and as an index of transmissibility. However, high heritability estimates along with high genetic advances are more helpful in predicting the gain under selection than heritability estimates alone. High heritability with high genetic advance was observed for all the biochemical traits under study, viz., total phenolics, CUPRAC, FRAP, ascorbic acid, and total flavonoids. Therefore, direct phenotypic selection would be more effective in improving these traits as the additive effect is more dominant. Similar findings have been reported by Kumar et al. (2013) and Nandi et al. (2021), emphasizing the need to include genetic variability and heritability for conducting any efficient breeding program. Because variability helps in selecting suitable lines and quantitative traits are mainly influenced by the environment, there should be a partition in terms of heritable and non-heritable components.

The expression of a character in a plant is the consequence of a chain of interrelationships between characters either directly or indirectly throughout other events. Hence, correlation coefficient analysis plays a significant role in checking the relationship between variables as well as in identifying genotypes with excellent and improved nutritional quality, and relevant studies help us to understand the suitability of various characters for indirect selection for one or more traits which results in a correlated response in several other traits (Searle, 1965). The positive and robust association observed between total phenolics and total flavonoids with the antioxidant assays CUPRAC and FRAP indicates that these correlations observed between the traits will help in the indirect selection of genotypes rich in antioxidant activity. Also, it was observed that the genotypes with high phenolics were potential candidates for scavenging free radicals, as reported by Kaur et al. (2014), Nandi et al. (2021), and Vanlalneihi et al. (2020), which included significant results in these similar traits. It was observed in the present study that total phenolics had significant and positive correlations with all the other traits suggesting that these relationships can be used as indirect indices for identifying genotypes with high antioxidant capacity.

It is possible to identify genotypes with high nutritional compounds through correlation analysis; however, dependence on the data generated by correlation studies does not provide a clear picture of the relationship of biochemical traits with other traits. It is because of the fact that in a given set of data, it is possible to keep into account only two sets of traits. Hence, to understand the relationships between different traits in a set of data, PCA analysis needs to be carried out to assist breeders in developing and identifying genotypes rich in nutritional compounds.

The chemometric approach is the chemical discipline that uses mathematical, statistical, and other methods engaging formal logic to design and select optimal measurement procedures and experiments and provide maximum relevant chemical information by analyzing chemical data. Various researchers have incorporated this approach to relate the correlation between different nutritional compounds and antioxidant capacity (Devi et al., 2015; Koley et al., 2019). Principal component analysis and agglomerative hierarchical clustering, two chemometric techniques, have been utilized in the present investigation to evaluate the eggplant’s bioactive compounds and antioxidant potential. Three major clusters were formed and explained with a heatmap, and it was evident that the genotypes in clusters 2 and 3 contributed more toward antioxidant capacity and can be used in future breeding programs. Multivariate analysis was also carried out to evaluate the genetic divergence among the various eggplant genotypes understudy to identify superior and diverse lines to be utilized for future nutritional breeding programs.

## Conclusion

From the above results, it is clear that a wide variability existed among the eggplant genotypes for different biochemical parameters and antioxidant activity. Earlier, the eggplant was thought to be a crop with no nutritional value; however, with this research work, it has become evident that this crop is rich in various bioactive compounds, and its consumption as a vegetable will have a beneficial effect on the human body owing to its antioxidant potential. Screening a large number of germplasm lines has led to the identification of high bioactive lines and provided scope for further improvement of the existing cultivated varieties. The high nutritional genotypes identified, viz., Pusa Krishna, HABI-2, EC 368225, G-5, and G-10, can be further used by eggplant breeders to identify and develop varieties/hybrids rich in nutritional quality. Furthermore, the wild species *S. insanum*, *S. khasianum*, and *S. xanthocarpum* can also be utilized as donor lines for carrying out nutritional breeding programs as they are the reservoir of many important biochemical genes.

## Data Availability

The original contributions presented in the study are included in the article/**Supplementary Material**. Further inquiries can be directed to the corresponding author.
