# Supplementary material for: Unveiling the nutraceutical potential of indigenous and exotic eggplant for bioactive compounds and antioxidant activity as well as its suitability to the nutraceutical industry
Source: Front Plant Sci. 2025 Feb 4;16:1451462. doi: 10.3389/fpls.2025.1451462 (PMC11832719; doi:10.3389/fpls.2025.1451462)
Supplement: Supplementary file 1 [file DataSheet1.docx]

**Supplementary Table 1.** Eigen values, % of variance and cumulative % of bioactive compounds and antioxidant activity among the eggplant accessions

| **Principal component** | **Eigen values** | **% of Variance** | **Cumulative %** |
| --- | --- | --- | --- |
| 1 | 3.084 | 61.68 | 61.68 |
| 2 | 1.021 | 20.44 | 82.12 |
| 3 | 0.429 | 8.60 | 90.72 |
| 4 | 0.311 | 6.22 | 96.94 |
| 5 | 0.153 | 3.06 | 100.00 |

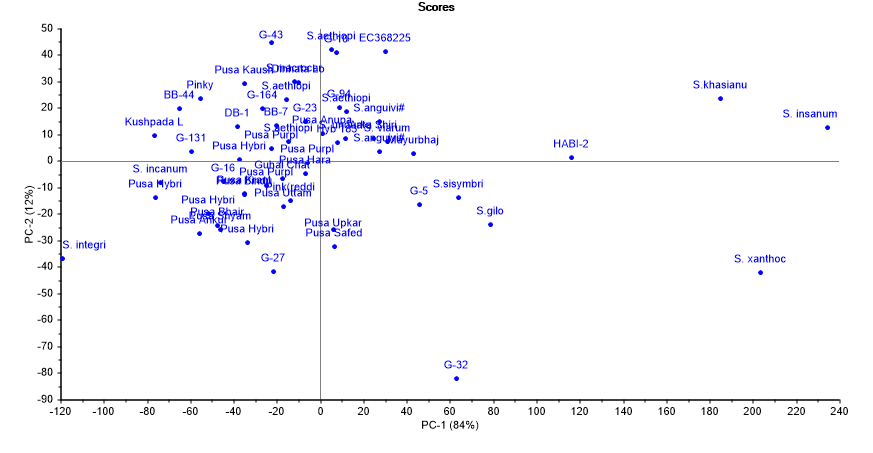


**Supplementary Figure 1.** Scree plot depicting eigen value of each principal component B Per cent variance explained by each of the five principal components C Variable correlation plot in the first two principal components

**Supplementary Figure 2.** Agglomerative hierarchical clustering (AHC) of 57 eggplant genotypes
