# Supplementary material for: Unveiling the nutraceutical potential of indigenous and exotic eggplant for bioactive compounds and antioxidant activity as well as its suitability to the nutraceutical industry
Source: Front Plant Sci. 2025 Feb 4;16:1451462. doi: 10.3389/fpls.2025.1451462 (PMC11832719; doi:10.3389/fpls.2025.1451462)
Supplement: Supplementary file 2 [file DataSheet2.docx]

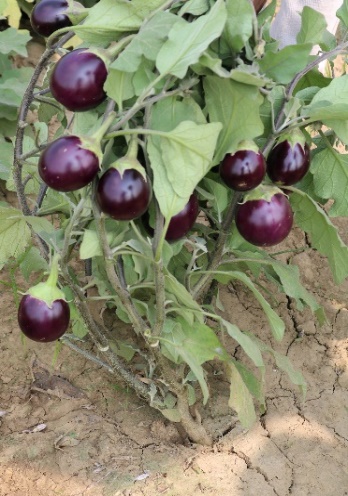

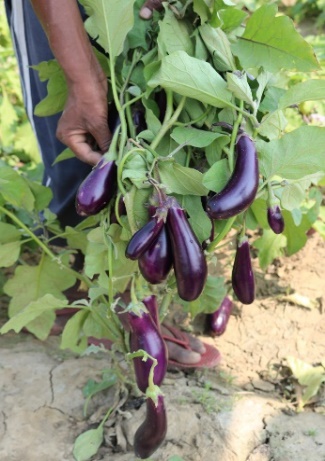

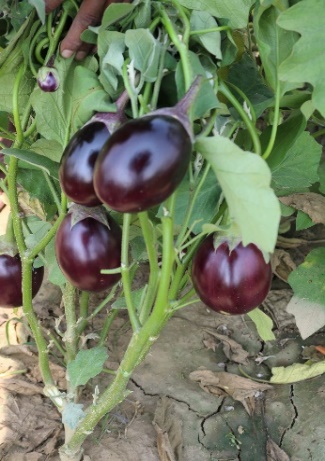

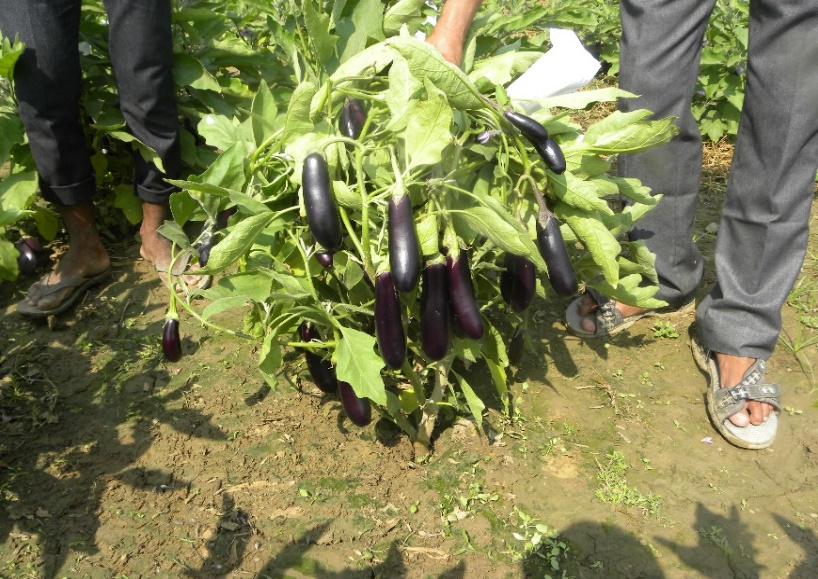

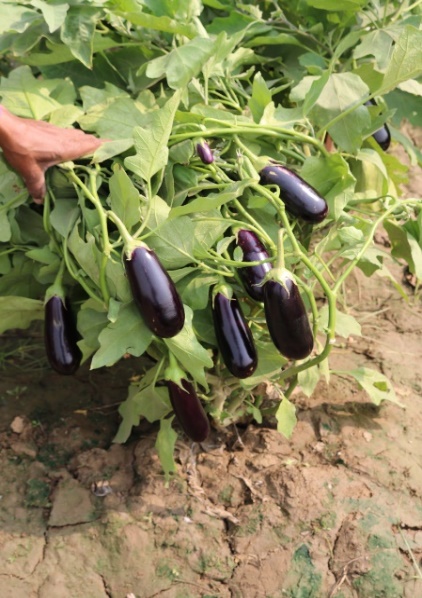

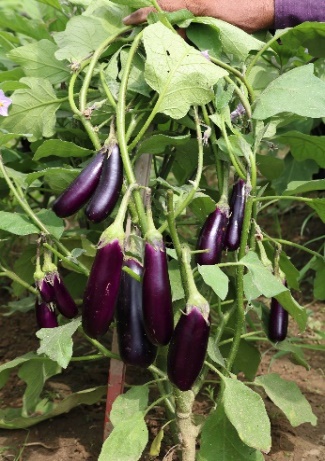


**1**

**2**

**3**

**4**

**5**

**6**


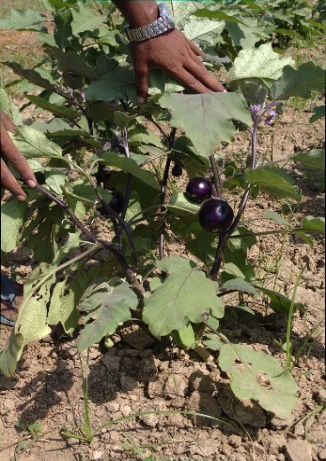

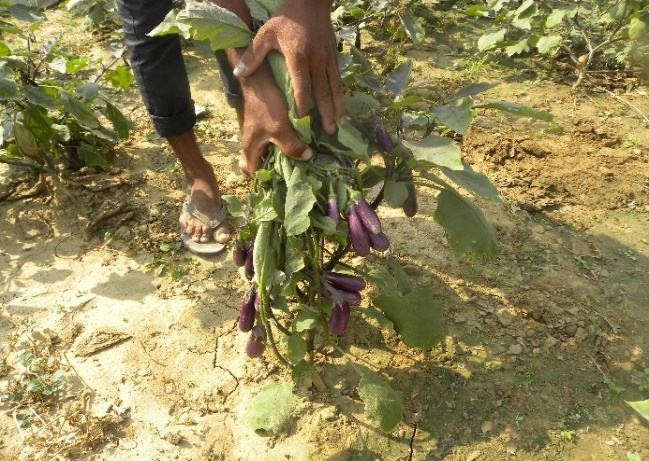

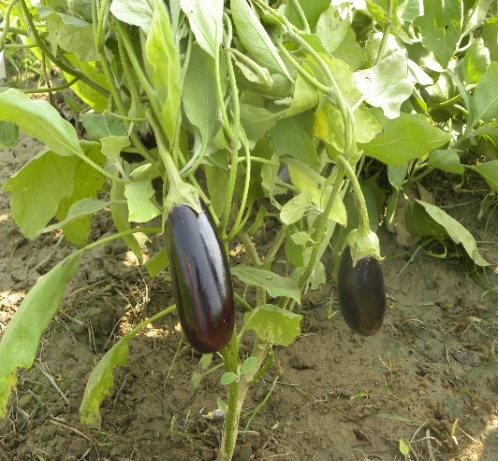

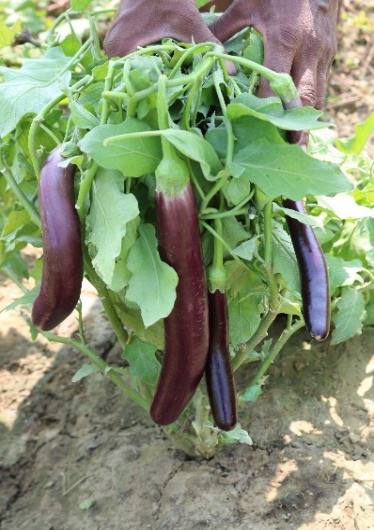

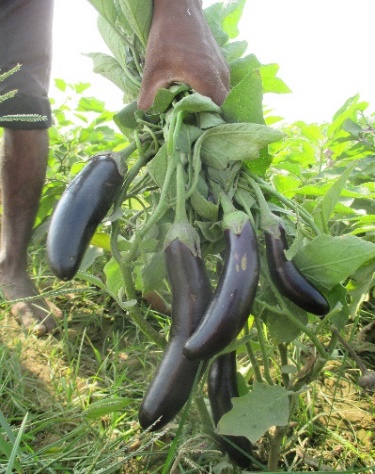

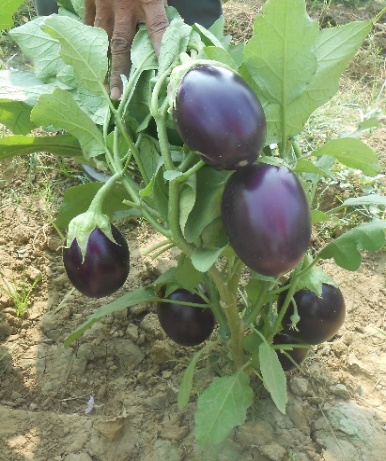


**7**

**8**

**9**

**10**

**11**

**12**

Supplementary FIGURE 1 A.

|  | Pusa Upkar | 7. | Pusa Bindu |
| --- | --- | --- | --- |
|  | Pusa Kaushal | 8. | Pusa Purple Cluster |
|  | Pusa Uttam | 9. | Pusa Bhairav |
|  | Pusa Shyamla | 10. | Pusa Purple Long |
|  | Pusa Kranti | 11. | Pusa Hybrid 5 |
|  | Pusa Hybrid 20 | 12. | Pusa Hybrid 9 |

**13**

**14**

**15**

**16**

**17**

**18**


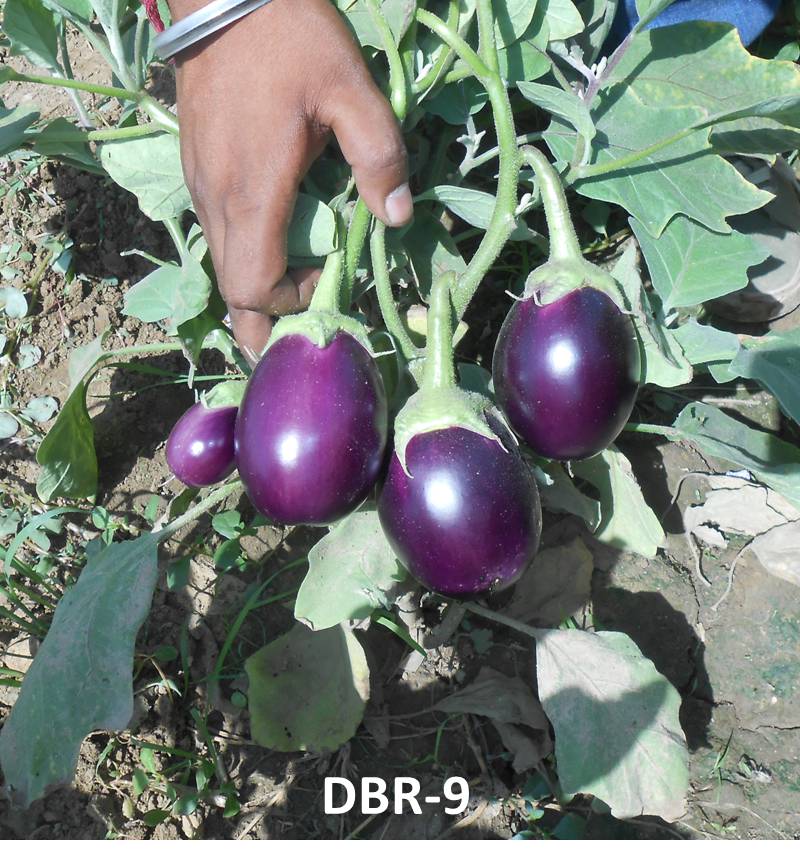

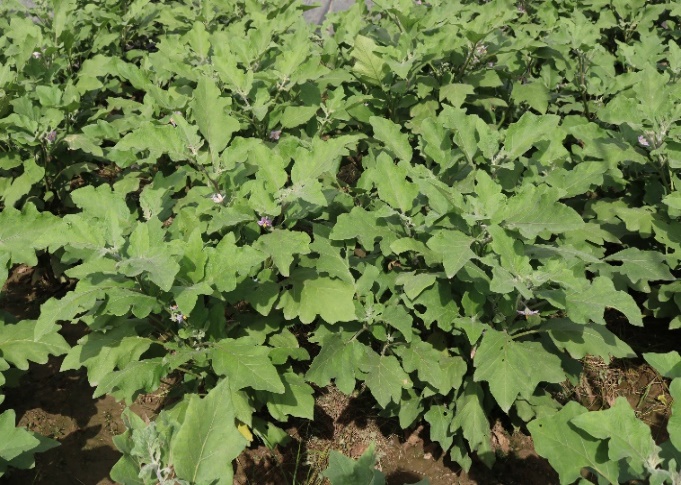

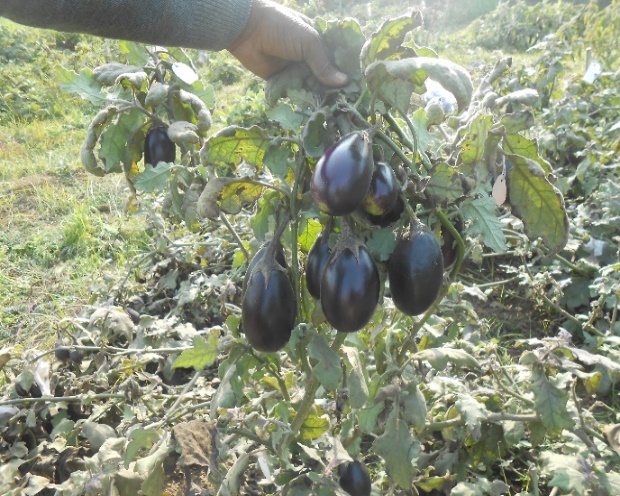


**19**

**20**

**21**

**22**

**23**

**24**


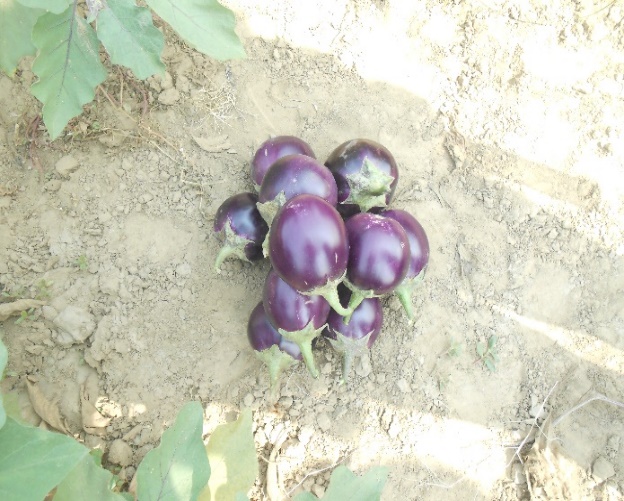

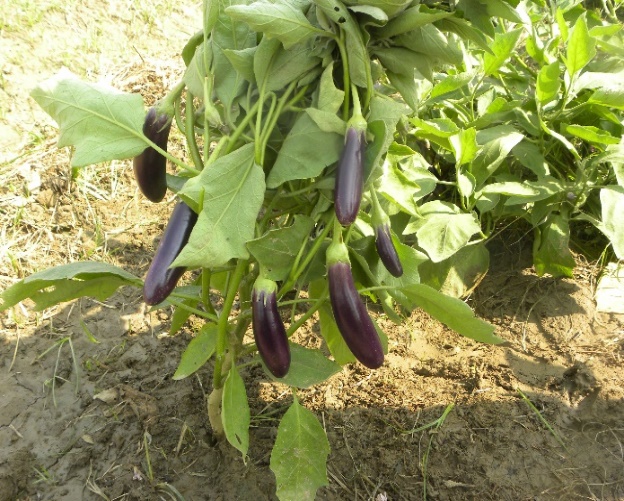

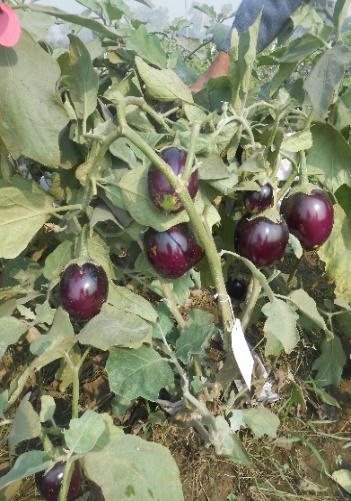

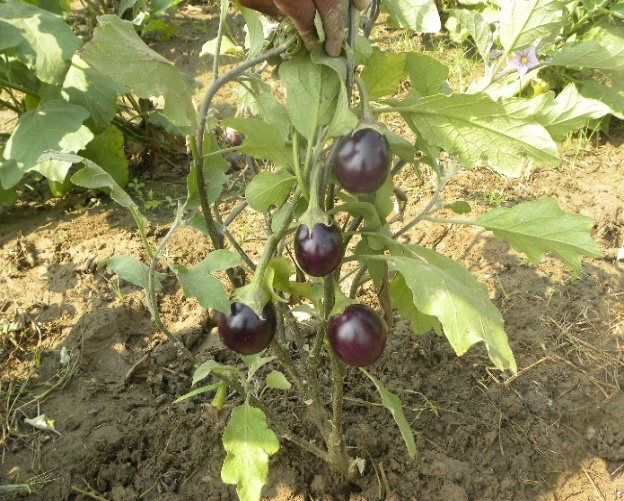

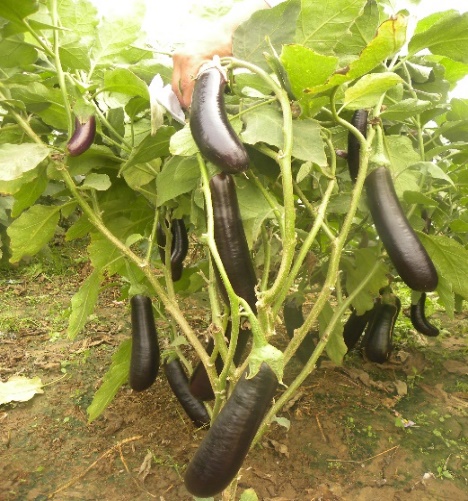

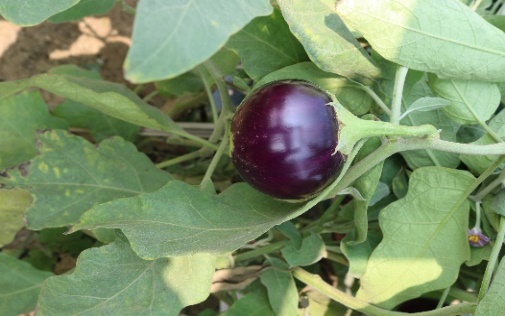

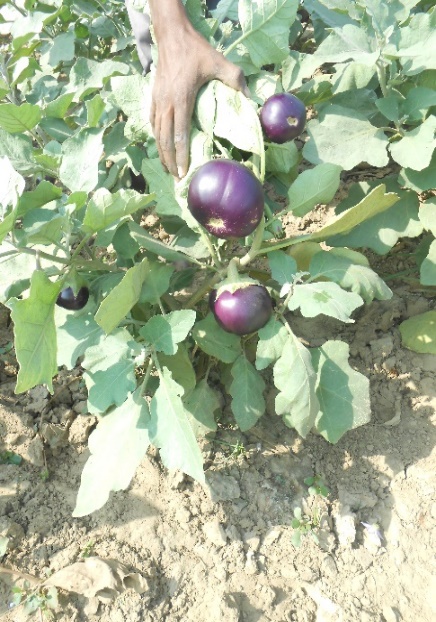

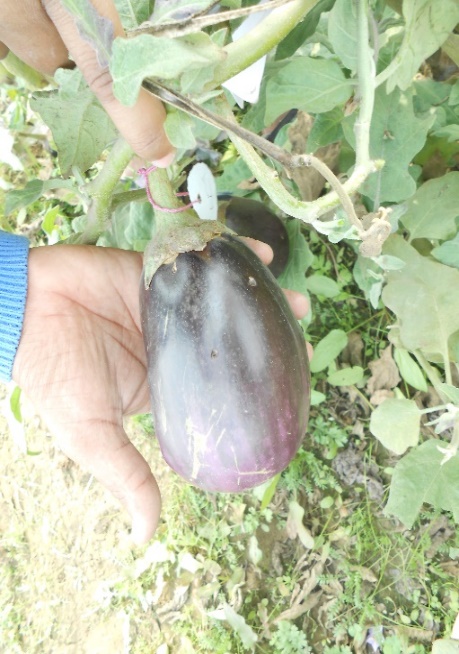

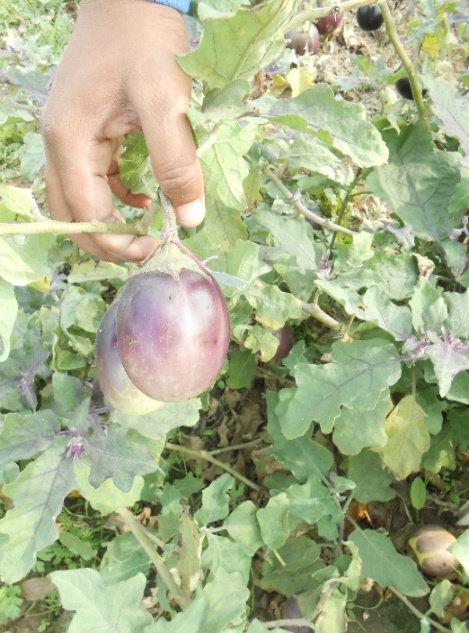


| 13. | Pusa Hybrid 6 | 19. | G-43 |
| --- | --- | --- | --- |
| 14. | Pusa Anupam | 20. | BB-7 |
| 15. | Pusa Purple Round | 21. | G-5 |
| 16. | Pusa Ankur | 22. | G-27 |
| 17. | Dinhata Local | 23. | Pusa Krishna |
| 18. | G-23 | 24. | G-94 |

Supplementary FIGURE 1 B.


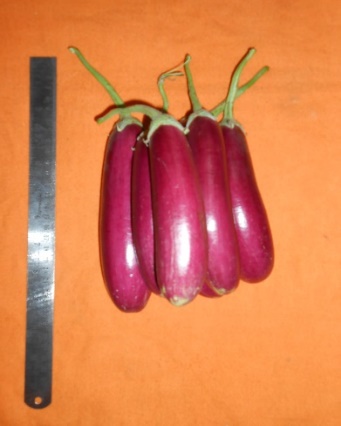

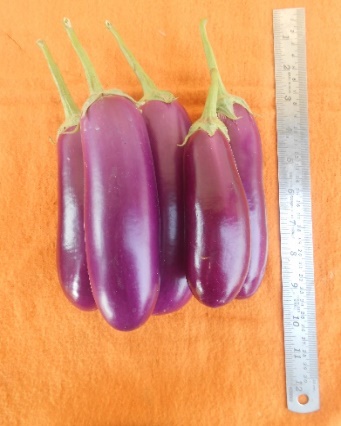

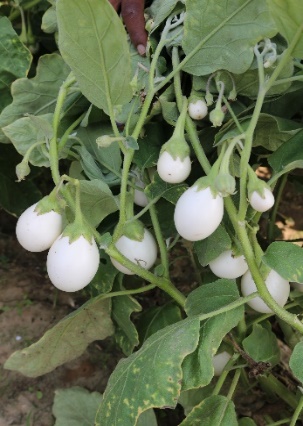

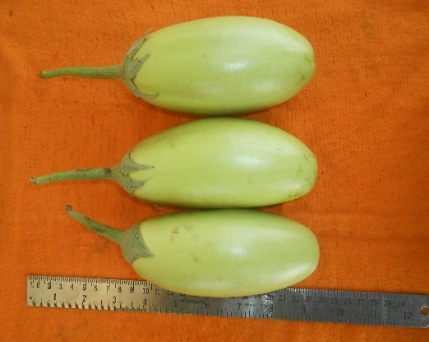

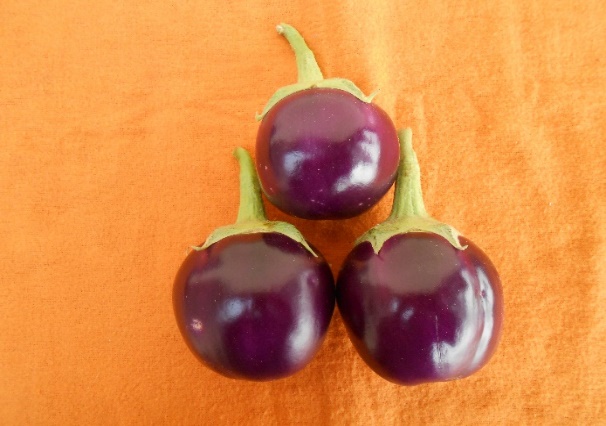

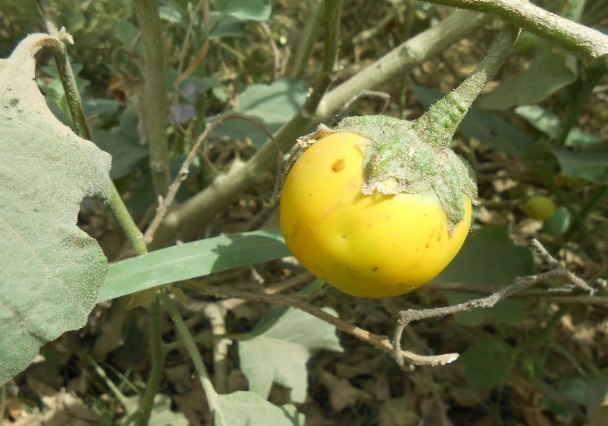


**25**

**26**

**27**

**28**

**29**

**30**


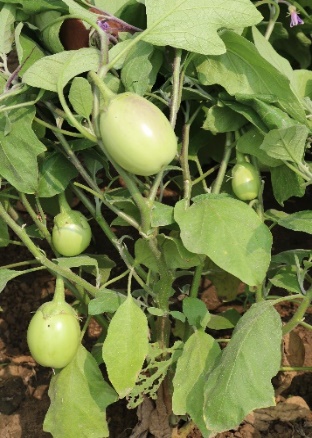

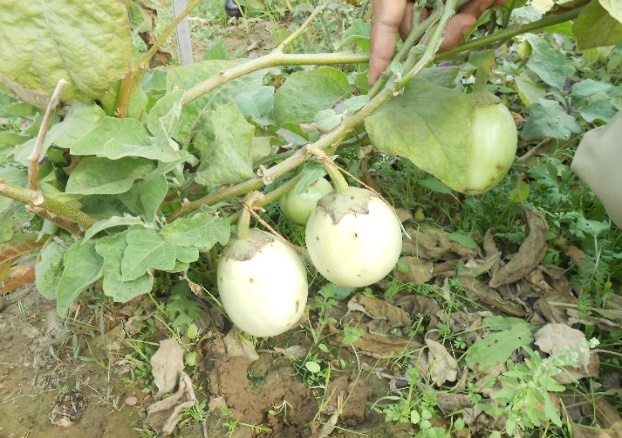

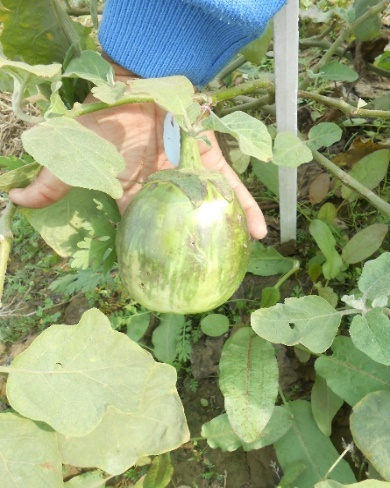

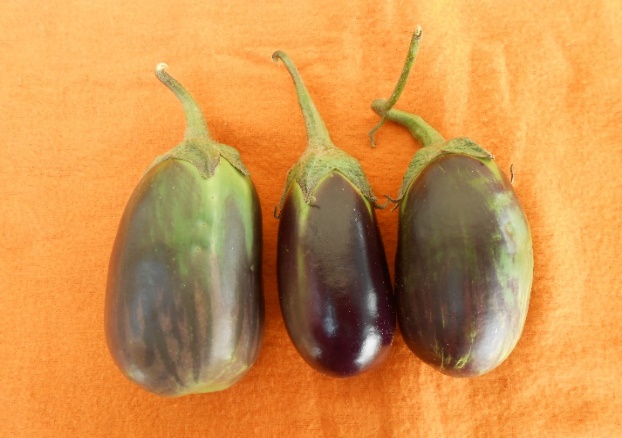

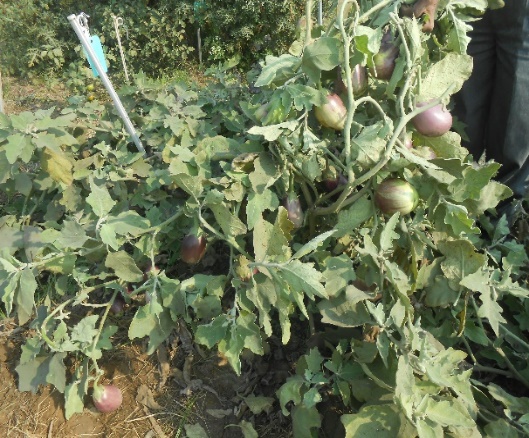

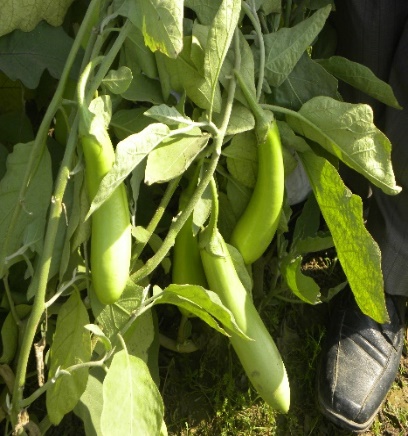


**31**

**32**

**33**

**34**

**35**

**36**

Supplementary FIGURE 1 C.

| 25. | Pink(reddish) | 31. | Pusa Hara Baingan 1 |
| --- | --- | --- | --- |
| 26. | Pinky | 32. | Kushpada Local |
| 27. | Pusa Safed Baingan 1 | 33. | Guhala Chatua Local |
| 28. | HABI-2 | 34. | Mayurbhanj Local |
| 29. | G-10 | 35. | BB-44 |
| 30. | *S. unduatum* | 36. | Hybrid 183 |


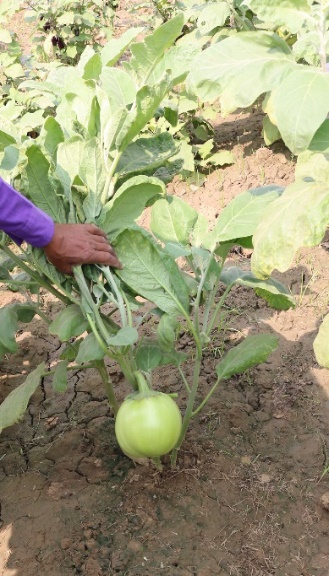

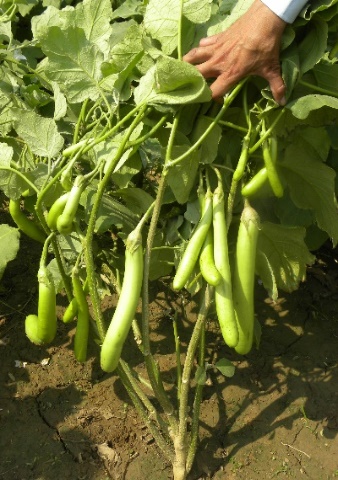

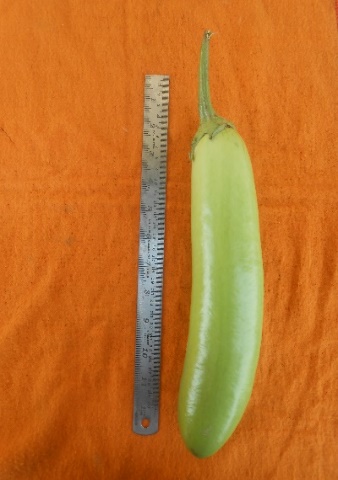

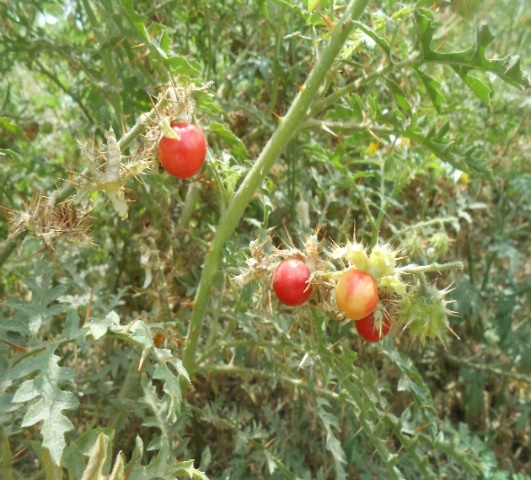

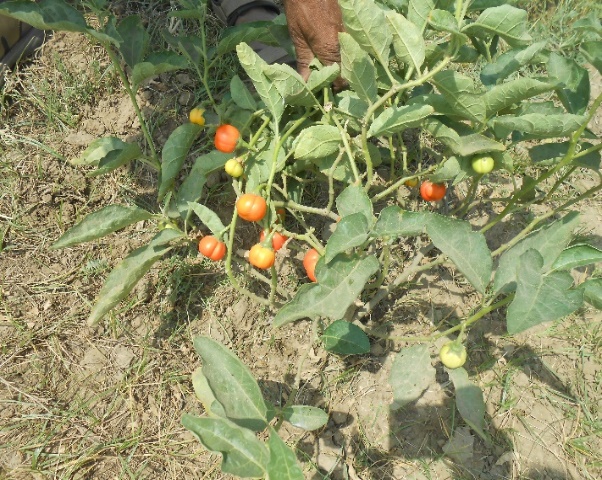

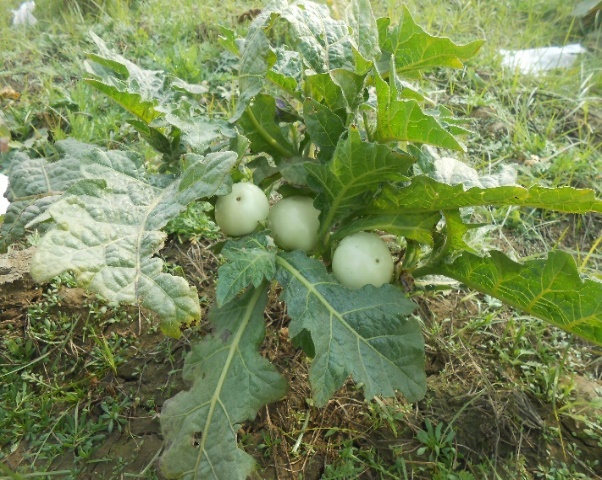


**37**

**38**

**39**

**40**

**41**

**42**


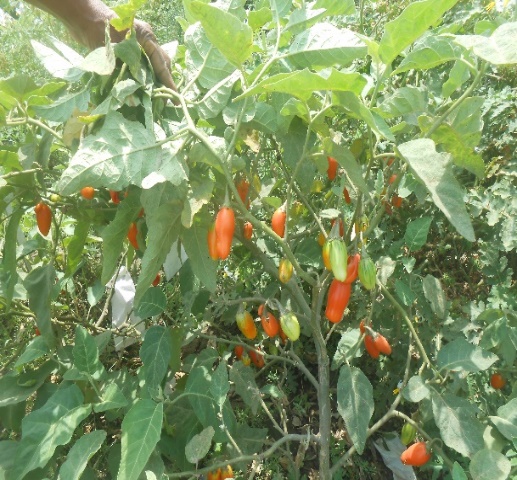

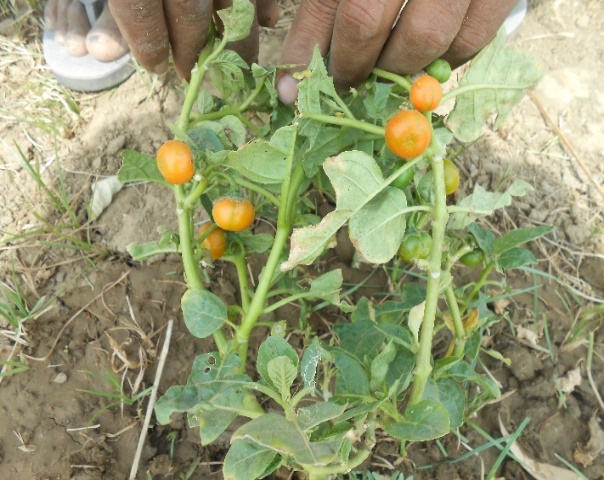

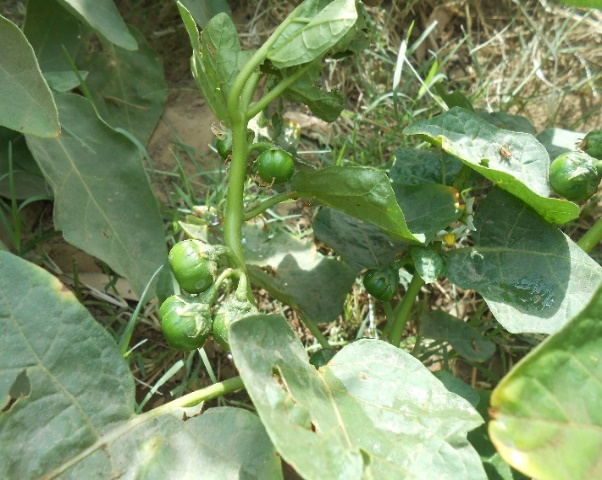

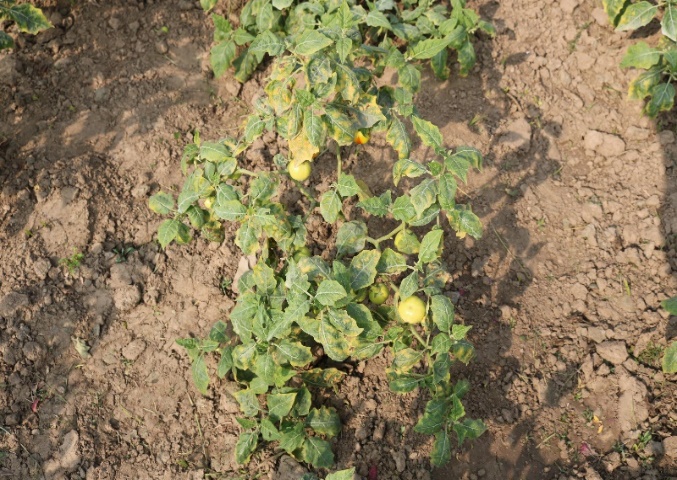

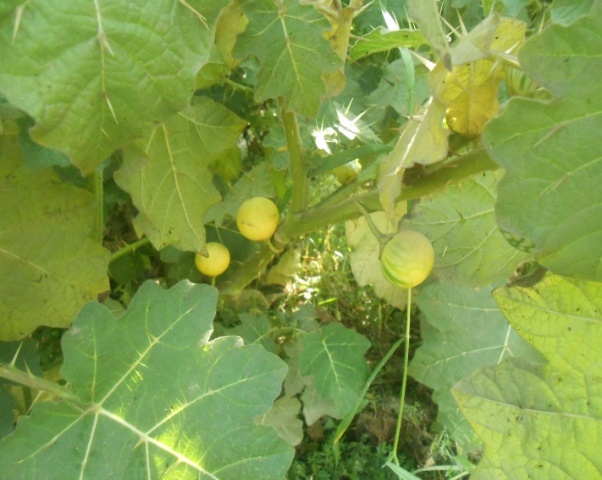

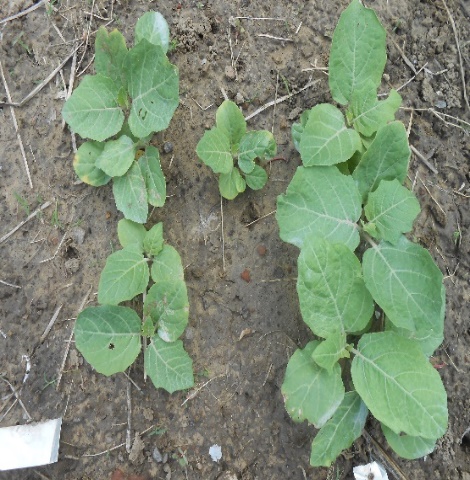


**43**

**44**

**45**

**46**

**47**

**48**

| 37. | G-131 | 43. | *S.aethiopicumAcc 2* |
| --- | --- | --- | --- |
| 38. | EC368225 | 44. | *S.anguiviAcc 1* |
| 39. | G-164 | 45. | *S.anguiviAcc 2* |
| 40. | *S.sisymbrifolium* | 46. | *S.aethiopicumAcc 3* |
| 41. | *S.aethiopicumAcc 1* | 47. | *S.aethiopicumAcc 4* |
| 42. | *S.macrocarpon* | 48. | *S. viarum* |

Supplementary FIGURE 1 D.


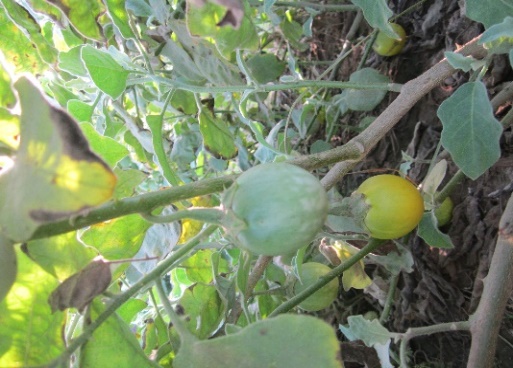

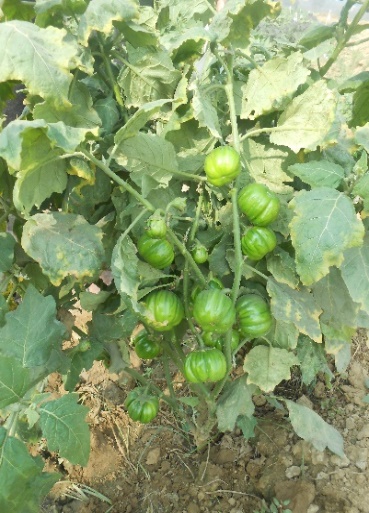

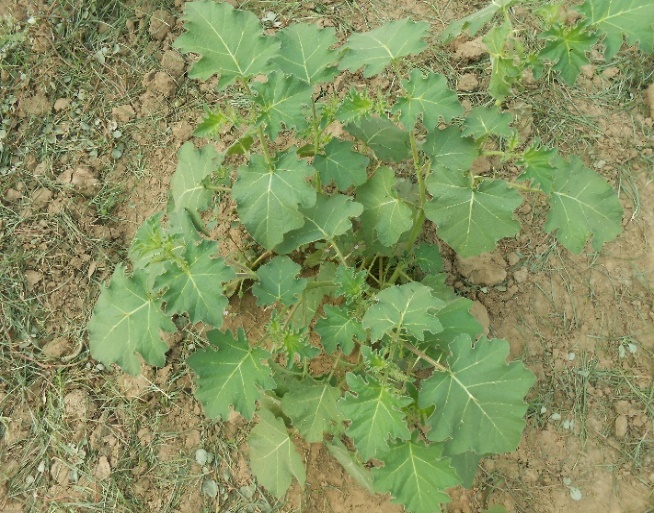

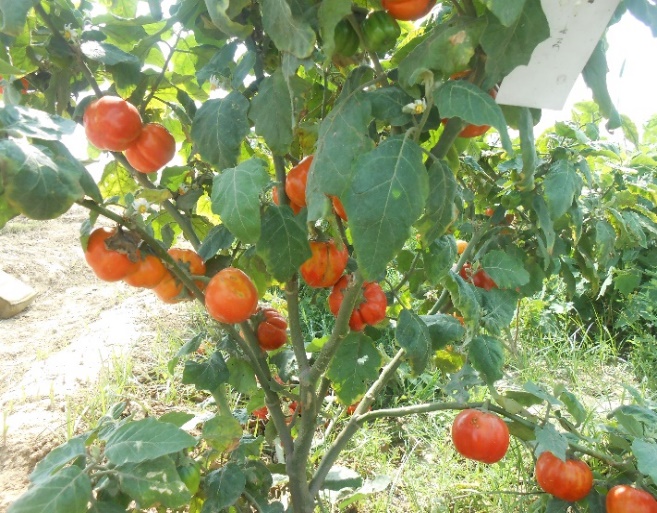

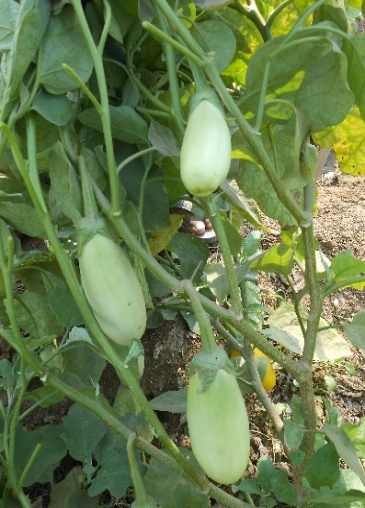


**49**

**50**

**51**

**52**

**53**


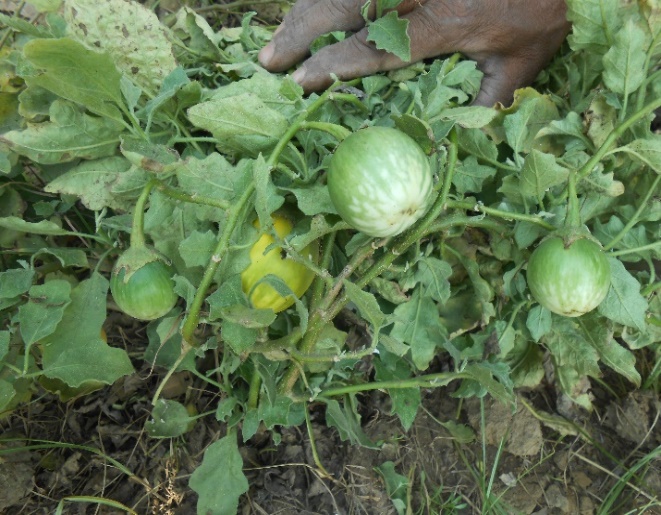

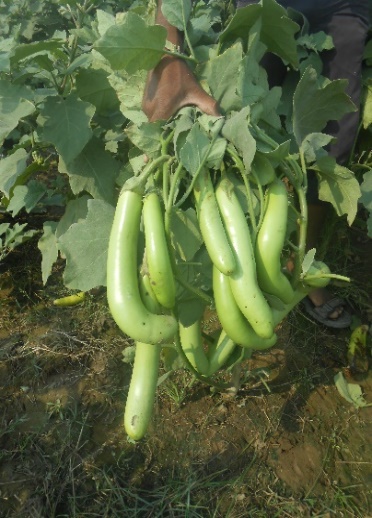

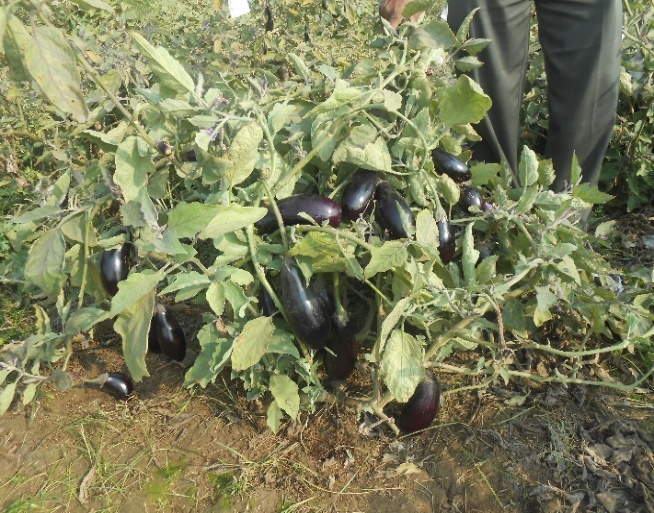

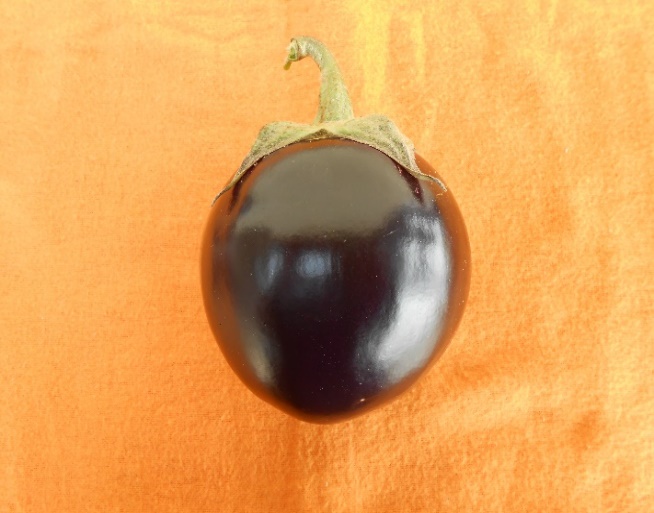


**55**

**56**

**57**

**54**

Supplementary FIGURE 1 E.

| 49. | *S. xanthocarpum* | 54. | *S. insanum* |
| --- | --- | --- | --- |
| 50. | *S.gilo* | 55. | Arka Shirish |
| 51. | *S.khasianum* | 56. | DB-1 |
| 52. | *S. integrifolium* | 57. | G-16 |
| 53. | *S. incanum* |  |  |
